# Adaptive Mutations in the JC Virus Protein Capsid Are Associated with Progressive Multifocal Leukoencephalopathy (PML)

**DOI:** 10.1371/journal.pgen.1000368

**Published:** 2009-02-06

**Authors:** Shamil R. Sunyaev, Alexey Lugovskoy, Kenneth Simon, Leonid Gorelik

**Affiliations:** 1 Division of Genetics, Department of Medicine, Brigham and Women's Hospital, Boston, Massachusetts, United States of America; 2 Harvard Medical School, Boston, Massachusetts, United States of America; 3 Departments of Drug Discovery, Biogen IDEC, Cambridge, Massachusetts, United States of America; 4 Department of Neurobiology, Biogen IDEC, Cambridge, Massachusetts, United States of America; Fred Hutchinson Cancer Research Center, United States of America

## Abstract

PML is a progressive and mostly fatal demyelinating disease caused by JC virus infection and destruction of infected oligodendrocytes in multiple brain foci of susceptible individuals. While JC virus is highly prevalent in the human population, PML is a rare disease that exclusively afflicts only a small percentage of immunocompromised individuals including those affected by HIV (AIDS) or immunosuppressive drugs. Viral- and/or host-specific factors, and not simply immune status, must be at play to account for the very large discrepancy between viral prevalence and low disease incidence. Here, we show that several amino acids on the surface of the JC virus capsid protein VP1 display accelerated evolution in viral sequences isolated from PML patients but not in sequences isolated from healthy subjects. We provide strong evidence that at least some of these mutations are involved in binding of sialic acid, a known receptor for the JC virus. Using statistical methods of molecular evolution, we performed a comprehensive analysis of JC virus VP1 sequences isolated from 55 PML patients and 253 sequences isolated from the urine of healthy individuals and found that a subset of amino acids found exclusively among PML VP1 sequences is acquired via adaptive evolution. By modeling of the 3-D structure of the JC virus capsid, we showed that these residues are located within the sialic acid binding site, a JC virus receptor for cell infection. Finally, we go on to demonstrate the involvement of some of these sites in receptor binding by demonstrating a profound reduction in hemagglutination properties of viral-like particles made of the VP1 protein carrying these mutations. Collectively, these results suggest that a more virulent PML causing phenotype of JC virus is acquired via adaptive evolution that changes viral specificity for its cellular receptor(s).

## Author Summary

JC virus is a highly prevalent human polyomavirus. Infection with this virus is generally benign and asymptomatic despite viral persistence in the kidney of many people. However, in immunocompromised individuals, very rarely, the infection can progress to become a potentially deadly brain disease called Progressive Multifocal Leukoencephalopathy (PML). The discrepancy between very high viral prevalence and low incidence of PML suggests that there could be some unique viral characteristics that regulate the progression from the asymptomatic infection to the PML. Identification of such factors will help us to understand the basis of PML development and hopefully will lead to the creation of new diagnostic and treatment tools for managing PML. In this work, we demonstrate that the part of the viral surface protein that is thought to be responsible for viral interaction with cellular receptors and infection acquires specific mutations that appear to be critical for the development of PML. These mutations are found more frequently than by simple chance and therefore are thought to be “positively selected.” Based on these results, we hypothesize that the specific mutations in the viral VP1 protein that we have identified are critical for the evolution of JC virus to the version associated with PML.

## Introduction

JC virus (JCV) is highly prevalent in the human population with over 70% of people showing anti-JCV antibody responses and up to 40% of the population displaying persistent viral shedding in the urine (reviewed in [1]). These epidemiological data indicate that the virus establishes chronic infection in a large fraction of the human population. Though normally asymptomatic, factors leading to immune deficiency, such as HIV or immunosuppressive drug therapy, can trigger an uncontrolled infection and replication of JCV in oligodendrocytes causing their death and resulting in progressive multifocal leukoencephalopathy (PML). Despite such a high infection rate and viral occurrence, JC virus causes PML in a very small fraction of immune deficient patients, including 4–5% of AIDS patients [2] and less than 1% of patients with lymphoproliferative diseases [3]. No pharmaceutical treatment option for PML currently exists and the only chance for patient survival is afforded by reconstitution of the patient's own immune response via HAART in AIDS or via drug tapering in pharmaceutically immunocompromised individuals. Identification of genetic and environmental risk factors influencing the development of PML is of great importance both for finding of therapeutic interventions and for the development of early diagnostic methods to help reducing the risks associated with immunosuppressive therapies.

Both host and viral genetics may contribute to PML. Earlier studies focusing on viral genetic factors identified duplications and rearrangements in the regulatory region of the viral genome [4]–[8]. Several studies also reported presence of several mutations in VP1 protein in the JC virus isolated from PML patients [8]–[10]. No comprehensive analysis of an association of changes in protein coding genes of JC virus with PML has been reported. Pathogenicity of viruses ranging from influenza virus [11],[12] to the mouse polyomavirus [13],[14], a close relative of human JCV, was shown to be determined by amino acid sequences involved in the binding of a viral capsid protein to sialylated glycan receptors. Changes in the affinity and specificity of the virus for its cellular receptor(s) affect viral infectivity and transmission, hence playing a crucial role in virulence. For example, a study of the mouse polyomavirus showed that VP1 amino acid changes rather than changes in the non-coding regulatory region are responsible for the increased pathogenicity of the virus [15],[16].

Consequently, we focused on VP1 protein and its relationship to PML. We relied on methods of molecular evolution to determine the presence of putative adaptive changes in the VP1 amino acid sequence associated with PML. The advantage of this approach over simple statistical association of sequence variants with the disease, is that it takes into account the phylogenetic relationship of viral strains and also allows identification of functionally significant amino acid positions by examining the rate of sequence evolution.

## Results/Discussion

**Figure 1:**
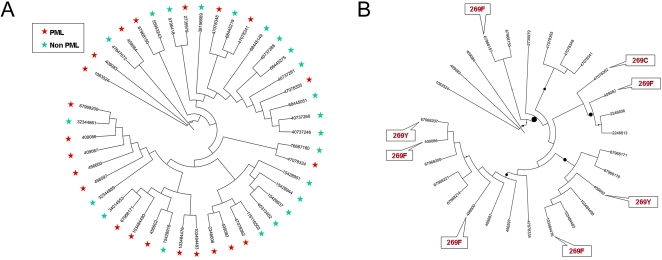
Phylogenetic distribution of PML associated viruses. (A) Broad phylogenetic distribution of PML causing JC viruses. Tree branches (labeled by GI numbers) corresponding to PML causing viruses are marked by red stars, viruses isolated from healthy subjects are marked by green stars. The tree is constructed based on DNA sequences of *VP1* gene using maximum likelihood method. Only one sequence per patient was included. (B) Phylogenetic distribution of mutations in the codon 269. The tree represents *VP1* genes (labeled by GI numbers) of viruses isolated from PML patients. Mutations in Ser269 codons are indicated by text inserts. Circles on branches reflect aLRT support. Position 269 was masked prior to constructing the tree to avoid attraction of branches with mutations of this codon.

JCV *VP1* gene sequences were downloaded from GenBank (Table S1) and used to construct a phylogenetic tree for a random subset of sequences isolated from healthy individual and full-length sequences isolated from distinct PML patients (Figure 1A). We used the PhyML maximum likelihood method [17] with F84 substitution model [18],[19]. Application of several methods incorporated in the PHYLIP package such as maximum likelihood method, distance-based and parsimony-based methods of phylogenetic reconstruction produced similar results. Viral sequences isolated from PML patients do not cluster on the phylogenetic tree and are broadly distributed among viral types and geographic origins of the samples (Figure 1A). This is further supported by the Slatkin-Maddison test for group separation (*p* = 0.38) [20]. In agreement with earlier studies [8],[21],[22], PML causing viruses are not limited to a specific viral phylogenetic type.

Next, we analyzed sequences from viruses isolated from PML patients as well as those from healthy subjects with the goal of determining whether PML associated evolutionary selective pressure is acting on the viral *VP1* gene. This analysis utilized the PAML package [23] designed to identify the presence of codons evolving under positive selection. PAML evaluates multiple evolutionary models using the parametric likelihood ratio test. We tested several models including a model of neutral evolution, a nearly neutral model allowing for purifying (i.e. negative) selection, and a heterogeneous model that allows some codon positions to evolve under positive selection and other codon positions to evolve under negative selection or neutrally (Table 1). We also tested a number of more complex models.

**Table 1:** Codons under positive selection in the PML sample.

*Mutations*			*Full length sequence set* (n = 28)	*Partial sequence set* codons 43–287 (n = 42)
			*P-value for the positive selection test*	
			2.5×10^−7^	3.5×10^−6^
*Position*	*WT*	*Mutant*	*Bayes Empirical Bayes posterior probability*	
55	L	F	0.82	0.94
60	K	M,E,N	<0.5	0.94
265	N	D,T	<0.5	0.85
267	S	F,L	0.80	0.92
269	S	F,Y,C	1.00	1.00

*VP1* sequences isolated from PML patients and random subsets of sequences isolated from healthy subjects were further analyzed using PAML [23]. We examined multiple models of sequence evolution incorporated in PAML including purely neutral model (M0), nearly neutral model (M1), model with positive selection (M2) and additional more complex models (M3–M8). We used likelihood ratio test (LRT) to compare the difference between models M1 and M2 to test for positive selection. P-values for positive selection in three datasets are shown together with Bayesian posterior probabilities for each codon position. Residues with Bayes Empirical Bayes posterior probabilities exceeding 0.5 are shown.

In the case of *VP1* sequences from JCV isolated from healthy subjects, the nearly neutral evolutionary model involving a mixture of neutrally evolving codons and codons under purifying selection clearly outperformed the purely neutral model (*p*-value 7.0×10^−6^). However, no statistical support was found for more complex models including models with positive selection. In contrast, for *VP1* sequences isolated from PML patients, allowing codons to evolve under positive selection resulted in a highly significant increase in the model likelihood (Table 1). The model with three categories of sites including sites evolving under purifying selection, neutral sites and sites under positive selection explained the data significantly better than the nearly neutral model limited only to neutral sites and the sites under purifying selection (*p*-value 2.5×10^−7^). More complex models did not show significant improvement over the simplest model with three categories of codons.

Four codon positions (corresponding to amino acids 55, 60, 267 and 269) were identified as evolving under positive selection in the PML sampling of full length sequences (Table 1). Bayesian posterior probabilities for positive selection computed by PAML were above 0.5 for these codon positions. The posterior probability for positive selection in codon 269 was close to 1. To increase the power of analysis, we added partial *VP1* sequences from JC virus isolated from PML patients. The addition of partial sequences revealed signal of positive selection in codon 265 (Table 1).

Interestingly, we never observed two *VP1* mutations in the same JCV isolate. Analysis by the Spidermonkey [24] method revealed epistatic interactions between positions 55 and 269 and between position 60 and 269 (with posterior probabilities 0.88 and 0.70 respectively). This may reflect “diminishing return” epistatic interactions, i.e. subsequent mutations are not beneficial and possibly detrimental on the background of a single mutation.

All substitutions in these five codons are clearly associated with PML. At least 52% of JC viruses (or 36 out of 69 sequences, including partial sequences) isolated from PML patients have at least one of these mutations, whereas none of these substitutions have been observed in 253 full length viral sequences from healthy subjects (Table S2). The strongest signal of positive selection in the PML sample was detected for the codon encoding amino acid at position 269. Figure 1B shows that multiple independent mutations of Ser269 to aromatic residues phenylalanine and tyrosine were observed in *VP1* from PML associated viruses. The existence of multiple independent mutations is not an artifact of phylogenetic reconstruction because lineages with mutant variants are separated by multiple branches with over 90% support by bootstrap analysis and support of the likelihood ratio test implemented in PhyML [17]. These lineages correspond to different, previously identified, phylogenetic types of JC virus and are from diverse geographic locations [21],[22].

**Figure 2:**
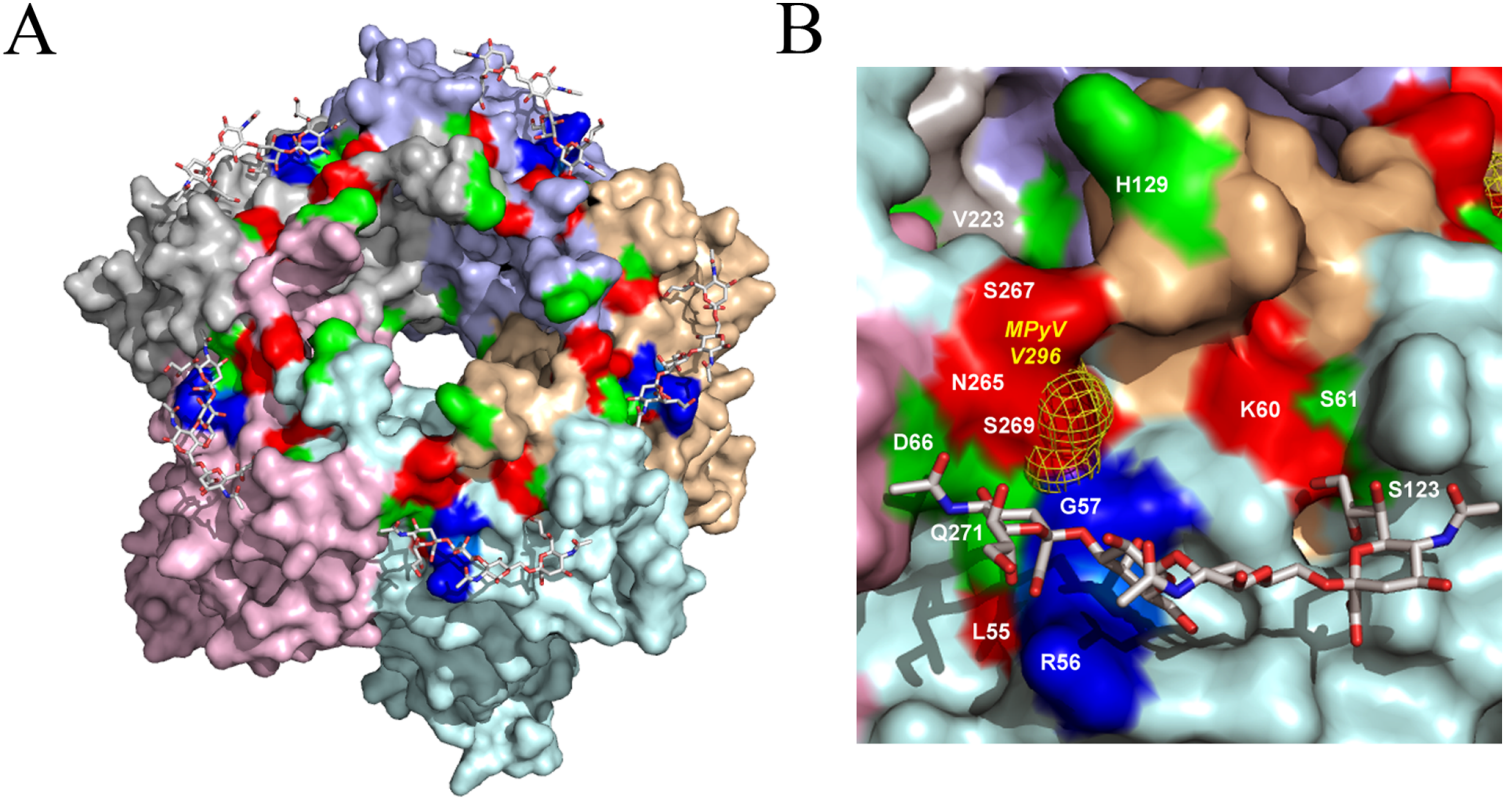
Structural model of JCV VP1/NeuNAc–(α2,3)–Gal–(β1,3)–[(α2,6)-NeuNAc]–Glc-NAc tetrasaccharide complex. (A) A model of JCV VP1 basic pentamer in complex with NeuNAc–(α2,3)–Gal–(β1,3)–[(α2,6)-NeuNAc]–Glc-NAc tetrasaccharide. Surfaces of five chains of JCV VP1 are shown in different colors. The RG motif essential for binding of core sialic acid is shown in blue. PML-associated mutated residues confirmed by PAML (Table 1) are shown in red (L55, K60, S265, S267, S269). Additional mutations unique to PML-isolated samples (Table S2) are shown in green (S61, D66, S123, H129, V223 and Q271). (B) A close-up view of NeuNAc–(α2,3)–Gal–(β1,3)–[(α2,6)-NeuNAc]–Glc-NAc tetrasaccharide/JCV VP1 complex. The color scheme is as described in panel (A). Location of V296 of MPyV VP1 which is predicted to be equivalent to S269 of JCV VP1 is shown in yellow mesh.

To get an insight into a functional role of the five identified amino acid positions, we constructed a three-dimensional molecular model of the JC virus VP1 bound to NeuNAc–(α2,3)–Gal–(β1,3)–[(α2,6)-NeuNAc]–Glc-NAc tetrasaccharide based on the crystal structure of MPyV VP1/oligosaccharide complex [25]. The structural model shown in Figure 2A suggests that all PAML-identified amino acids are clustered on the surface of the VP1 protein at the sialic acid binding site and are likely to be involved in sialic acid binding. Additionally, we predicted that L55F, K60M, S267F, and S269F substitutions may induce steric clashes with the modeled saccharide leading to a decrease in the affinity of the interaction. Affinity to sialic acid was related to viral pathogenicity in multiple studies of flu virus, mouse polyomavirus, and mouse minute virus [11]–[14],[26]. Particularly, pathogenicity of mouse polyomavirus, a close relative of the JC virus, was mapped to a VP1 amino acid substitution at position 296 [13], a position orthologous to position 269 in human JC virus that showed the strongest signal of positive selection in PML-causing viral isolates in our study. As shown in Figure 2B, serine 269 of the human JC virus and valine 296 of the mouse polyomavirus occupy identical locations in the sialic acid binding pocket.

We note that positions 61, 66, 123, 129, 223 and 271 are all limited to the PML sample (Table S2) and also line up with the sialic acid binding pocket (Figure 2B). It is possible that those residues went undetected by the PAML analysis due to the small sample size and that the development of PML is accompanied by positive selection for amino acids involved in sialic acid binding in a majority of cases. The length of the phylogenetic tree in our analysis is short thus limiting power to detect positive selection [27],[28]. Likelihood ratio test for detecting positive selection using a short tree is conservative [27], and Bayes Empirical Bayes analysis is of limited power [28]. Thus, additional PML-specific VP1 mutations can also be positive selected. Mutations at residue 107 are also found exclusively in the PML sample. However, it did not show evidence of positive selection according to PAML and is not located in the sialic acid binding pocket.

In order to experimentally verify the role that these substitutions play in sialic acid binding by the VP1 capsid, we recombinantly produced viral like particles (VLP) from VP1 protein encoded by several different naturally occurring viruses. We generated VLPs from viral *VP1* sequences encoding substitutions with one of the two strongest signals of positive selection identified by PAML, one with phenylalanine at position 269 (F269) and another one with phenylalanine at position 55 (F55). As controls we used two different *VP1* genes that do not harbor any of the identified PML-associated mutations, one from a healthy individual (WT) and another one from a PML patient (Mad-1) (Table S3). Viral hemagglutination of red blood cells (RBCs) has been shown to be a reliable measure of sialic acid binding by polyomaviruses [16],[29]. We tested all four VLPs in a hemagglutination assay. Strikingly, both F55 and F269 variants displayed more than 8000-fold lower HA activity than either control VLP (Table 2). Specifically, the F55 variant completely failed to agglutinate human type O RBCs even at 200 µg/ml, the highest concentration tested, and the F269 variant displayed very low HA activity as it caused hemagglutination only at concentrations above 25 µg/ml. At the same time both L55 and S269 carrying variants (WT and Mad-1) caused hemagglutination of RBCs at concentrations down to 0.375 ng/ml and 6.25 ng/ml, correspondingly. We note that the F55 mutant has the single amino acid difference with its corresponding wild type variant (WT). Therefore the change in hemagglutination can be specifically attributed to this amino acid replacement. In addition to the change in position 269 the F269 mutant variant has two additional amino acid positions that are different from its corresponding control variant (Mad-1). Both of those amino acid changes are not PML specific (Table S3 and Table S2) and are unlikely to explain the difference in hemagglutination. While the Mad-1 isolate had originated from a PML patient [30] it does not contain any of the PML-specific mutation which correlates well with its ability to hemagglutinate RBCs. The lack of PML-genic mutations in this PML isolate suggests that VP1 mutations are not an exclusive mechanism leading to PML development.

**Table 2:** Residues 55 and 269 in VP1 protein play very important role in hemagglutination of RBCs by Viral Like Particles (VLPs).

Viral variant	Minimum HA VLP concentration, ng/ml
WT1	0.08
55F	>200,000
WT2 (Mad-1)	6.25
269F	50,000

Hemagglutination was conducted as described in Materials and Methods using serial dilutions of VLPs starting from 200 µg/ml. VLPs were added to type O RBC and incubated at 4°C for 3 hours. Agglutination is visualized by the lack of a round pellet formed by the settling of RBCs out of suspension. F55 is a VP1 variant with phenylalanine at the position 55 (AAT09831), F269 is a VP1 variant with phenylalanine at the position 269 (BAE0011). WT (AAQ88264) and Mad-1 (P03089) are VP1 variants with leucine and serine at positions 55 and 269 respectively.

Although we do not know at the moment how these amino acid substitutions affect viral infectivity *per se*, it is reasonable to assume that a virus harboring such substitutions is adequately infectious as it was sufficiently abundant in the CNS of PML patients to be isolated. Therefore, it is tempting to speculate that changes in glycan specificity would allow JCV to loose its specificity to sialated glycans expressed outside of the CNS (e.g. RBCs). Thus, such a virus would avoid getting trapped on “pseudoreceptors” in the periphery and travel unhindered from sites of viral shedding to enter the brain. Mutated virus must still maintain its specificity to glycans expressed on oligodendrocytes. This would be consistent with the observation from the mouse polyomavirus model where a mutation in a position orthologous to position 269 of JCV affected viral ability to bind RBCs and also lead to the dramatic increase in viral dissemination through the animal with a lethal outcome [15],[16]. Furthermore, there are several reports of JCV detection in tonsils of many asymptomatically infected individuals [31],[32]. Although this observation was taken as a support for the JCV infection of tonsil cells, it could be alternatively explained by the viral trapping in lymphoid tissues. That would be consistent with JCV binding to sialic acid in the tonsil tissue [33].

An alternative but not mutually exclusive hypothesis would be that PML associated *VP1* mutations increase JCV tropism for brain white matter cells leading to the increased viral infectivity and replication in oligodendrocytes. Finally, another non-mutually exclusive explanation of the role these mutations in PML might be immune-escape by the virus. It is theoretically possible that out of the polyclonal immune response directed against the VP1 molecule only a limited number of antibodies directed against the cell receptor binding site (i.e. sialic acid) would provide protection against the spread of the viral infection. Mutation of an amino acid within an epitope crucial for the protective immunity could allow virus to bind to its target cells and spread uninhibited. Given the large number of mutations that are specific for PML it is likely that not a single mechanism but rather a multiplicity plays a role in PML etiology in different PML cases.

How do these mutations occur in PML and why, despite a very high prevalence of JCV, do only a small proportion of immune deficient patients develop PML? Absence of clustering of the mutations on the viral phylogenetic tree suggests that they arise independently in individual patients rather than persist in the general populations as pathogenic viral variants. It is worth noting that this hypothesis appears to be strongly supported by the original observation of Loeber and Dorries [6] where the investigators reported the isolation of two viral strains from kidney and brain of the same PML patient. The genome of the virus isolated from the brain was almost identical to that isolated from the kidney with two exceptions; presence of phenylalanine instead of leucine in position 55 and a rearrangement of the regulatory region. Previously no significance could be attached to the L55F mutation and that observation led to the generation of the hypothesis on the sole importance of viral control region rearrangement in “PML-genic” adaptation of the virus. Based on our findings we would like to propose that *VP1* mutations play a very significant role in the mechanism of PML emergence. Once a specific mutation affecting sialic acid binding occurs it allows virus to spread to the brain and infect oligodendrocytes. The fact that the mutant virus was not detected in the kidney [6] may suggest that that particular change in glycan binding does not offer any selective advantage to the mutated virus in kidney. The mutations might have occurred and hence allowed the virus to establish the residence in the brain under the conditions of immune suppression shortly or long before the PML. Since no viral replication was detected in brains of asymptomatic individuals we believe it is unlikely that compartmentalized evolution (i.e. intra CNS) prior to PML development could account for the presence of mutated VP1 in CNS of PML patients. However, the issue of JCV latency in normal brain still remains controversial so it is still formally possible that non-mutated virus had entered the brain and mutations arose in the brain and not periphery, e.g. kidney.

It appears that the healthy immune system effectively controls viral activation in the brain. However, as soon as the immune system fails in the misfortunate individual harboring such a mutated virus, the virus begins actively proliferating in oligodendrocytes causing PML. It is also possible that a healthy immune system efficiently suppresses newly developed mutants in their peripheral site (e.g. kidney) and prevents them from spreading and infecting new target cells. Thus the timing of PML development may be mutation limited and the interplay with environmental or host genetic factors contributed to the non-deterministic development of PML. Alternatively, PML development may be controlled by interactions of *VP1* mutations with additional genetic alterations of the virus including rearrangement of the viral regulatory region as it might give the virus additional selective advantage in increasing viral replication in oligodendrocytes.

Altogether our findings suggest that JCV *VP1* mutations affecting its receptor specificity may be responsible for PML pathology. These results pave the way for the discovery of novel anti-polyomavirus therapeutics and diagnostics of diseases caused by these viruses. The exact role that these mutations play in etiology of PML as well as how and where they arise requires further extensive investigation that would involve VP1 sequence analysis of longitudinal and time matching samples from different organs (e.g. urine, blood, CSF) and from a variety of PML patients.

## Materials and Methods

### Sequence Analysis

35 full length *VP1* sequences of JC viruses isolated from PML patients and 253 full length *VP1* sequences of JC viruses isolated from healthy subjects were downloaded from Genbank. In addition, 20 partial *VP1* sequences were available from Genbank enabling the analysis of the total of 55 sequences for positions 43–287. In addition to these 55 VP1 sequences isolated from PML patients Table S1 also contains information from twelve more partial sequences available from a publication by Sala et al. [34]. We note that all viral samples isolated from PML patients originated from brain or CSF tissues except one sample isolated from kidney (Table S1). All viral samples isolated from healthy subjects originated from urine. Multiple sequence alignments were constructed using TCoffee [35]. A number of PML sequences were isolated from the same individual. Since we were studying evolution of viral sequences we accepted same patient isolated sequences for our analysis as long as they differed from each other by ≥1 nucleotide. However, we excluded identical “clonal” sequences from our analysis. This resulted in the final set of 28 full-length *VP1* sequences and 42 partial *VP1* sequences isolated from PML patients. All information on the origin and clonality of sequences is contained in Table S1. Phylogenetic trees were built using the PhyML maximum likelihood method [17] with F84 substitution model [18],[19] and using several methods included in the PHYLIP package (Felsenstein, J. 2005. PHYLIP version 3.6. *Distributed by the author*. *Department of Genome Sciences*, *University of Washington*, *Seattle*). *VP1* sequences isolated from PML patients and random subsets of sequences isolated from healthy subjects were further analyzed using PAML [23]. We examined multiple models of sequence evolution (M0–M8). We used likelihood ratio test for difference between models M1 and M2 to test for positive selection. Residues with Bayes Empirical Bayes posterior probabilities exceeding 0.5 in the analysis of either full-length or partial set are reported in Table 1. We used Spidermonkey [24] to analyze epistatic interaction. Spidermonkey was run through the Datamonkey web server [36]. Slatkin-Maddison test was used to evaluate separation of PML-casing JC viruses and JC viruses isolated from healthy subjects [20]. We used HyPhy package to compute the Slatkin-Maddison test [37]. The significance of group separation was determined using the permutation test (1000 permutations).

### Hemagglutination Assay

Hemagglutination assay was performed as previously described [38],[39]. Briefly, human type O blood was washed twice and suspended in Alsever's buffer (20 mM sodium citrate, 72 mM NaCl, 100 mM glucose, pH 6.5 adjusted with acetic acid) at a final concentration of ∼0.5%. Serial two-fold dilutions of VLPs were prepared in Alsever's buffer and an equal volume of RBCs was added into each well of a 96-well “U” bottom microtiter plate and incubated at 4°C for 3–6 hr. Minimum HA concentration is the lowest concentration of VLP protein that still agglutinated RBCs.

### Viral-Like Particles

Genes encoding the VP1protein from JC virus strains BAE00117, AAT09831 and AAQ88264 were created synthetically and cloned into the Gateway pDEST8 (Invitrogen) shuttle vector for transfer into the pFASTBAC baculovirus expression system for baculovirus expression in SF9 cells. Purification of VLPs was performed from roughly 100 grams of frozen cell pellets from 5 liters of culture. Cells were resuspended in 500 ml of PBS containing 0.1 mM CaCl_2_. The cells are disrupted by passing the cell suspension twice through a Microfuidics Microfluidizer. Cell debris was removed by pelleting at 8000×G for 15 minutes. The supernatant volume was adjusted to 720 ml with PBS/CaCl_2_ and loaded onto 5 ml 40% sucrose cushions. Virus-like particles were twice pelleted through the sucrose cushions in a SW28 rotor at 100,000×G for 5 hours. The VLP pellets were resuspended in PBS/CaCl_2_ and then treated with 0.25% deoxycholate for 1 hour at 37°C followed by the addition of 4 M NaCl/0.1 mM CaCl_2_ for 1 hour at 4°C. Precipitated material was removed by centrifugation at 8000×G for 15 minutes. The resulting supernatant was concentrated and buffer exchanged by ultrafiltration through a Pelicon-2 500,000 MWCO membrane (Millipore). The concentrated VLPs were applied to the center of a 25–40% step gradient of Optiprep (Sigma) and banded at 190,000 g for 17 hours in a type 50.2 rotor. VLP bands were collected and then concentrated and buffer exchanged in an Amicon stirred cell with a 300,000 MWCO membrane. VLP quality was determined by gel electrophoresis and electron microscopy (Figure S1). Protein concentration was determined by the Micro BCA assay (Pierce). Electron microscopy was performed at the Department of Cell Biology at Harvard Medical School. VLP samples were placed on carbon grids, briefly washed in water and negatively stained with uranyl acetate and allowed to dry. The grids were viewed and imaged on a Technai G2 Spirit BioTWIN TEM.

### Molecular Modeling of JCV VP1/Tetrasacharide Complex

A homology model of the JCV VP1 protein pentameric unit was built with MODELER [40] using the structure of MPyV VP1 (Protein Data Bank ID: 1VPS [25] as a template. The model of NeuNAc–(α2,3)–Gal–(β1,3)–[(α2,6)-NeuNAc]–Glc-NAc tetrasaccharide was build based on the structure of NeuNAc–(α2,3)–Gal–(β1,3)–[(α2,6)-NeuNAc]–Glc-NAc bound to MPyV VP1 [25]. The model of the JCV VP1/NeuNAc–(α2,3)–Gal–(β1,3)–[(α2,6)-NeuNAc]–Glc-NAc tetrasaccharide was extensively refined in CHARMM [41] and was analyzed using PyMOL visualization software (The PyMOL Molecular Graphics System (2002) DeLano Scientific, Palo Alto, CA, USA. http://www.pymol.org).

### Accession Numbers

The National Center for Biotechnology Information (NCBI) (http://www.ncbi.nlm.nih.gov/sites/entrez?dbprotein) Protein database accession numbers for JCV VP1 sequences from non-PML patients.

BAC66394, BAC66418, BAC66382, BAB11716, BAB11722, AAK28466, AAK28460, AAK28478, BAC66400, BAC66388, BAD06126, BAC66406, AAK97970, AAK97964, BAC81952, BAC81958, AAM89309, AAM89303, BAC81922, BAC81916, BAC81910, BAC81904, AAM89297, BAD11896, BAC81946, BAE45426, BAE45360, BAD06120, BAE45432, BAA01962, BAE45420, BAE45414, BAE45384, BAE45378, BAE45372, AAK98036, BAE45402, BAE45408, BAE45396, BAE45444, BAD06024, BAE75838, BAE75832, BAE75826, BAE75820, BAE75814, AAK98030, AAK98024, AAK98018, AAK98010, AAK98006, AAK98000, BAE45438, BAE45390, BAD06108, BAD06102, BAD06096, BAD06090, BAD06084, BAD06048, BAD06030, BAD06018, BAD06054, BAD06042, BAD06036, AAG30857, BAE45366, AAN85455, BAD06150, AAN85449, AAK98042, BAD06174, BAD06156, BAD06072, BAD06060, AAN85473, BAC81840, BAF40841, BAF40835, BAF40829, BAF40823, BAF40811, BAF40847, BAF40781, BAF40817, BAF40799, BAF40793, BAF40787, BAF40745, AAN85467, AAN85461, BAC81834, BAF40751, BAF40805, BAA01961, BAD98972, BAD98966, BAD06227, BAC66430, BAC66412, BAD91887, BAD21235, BAD27118, BAC66424, BAA01958, BAB11710, BAD21265, BAD21259, BAD21253, BAD21241, BAD21229, BAD21247, BAD21283, BAD21271, BAD21295, BAD21289, BAA01959, BAA01960, BAD11848, BAD11842, AAM89339, AAM89327, BAD11836, BAC81852, BAC81858, BAD06144, AAG37198, AAM89315, BAD06138, BAD11890, BAD11884, BAD11878, BAD11872, BAD11866, AAK97994, BAB11698, BAC81940, BAC81964, AAK97946, BAD06066, BAF40769, BAC81870, BAC81864, BAC81934, BAC66376, BAC81874, BAC81846, AAK97940, BAC81898, BAC81892, AAK97922, AAK97916, AAK97910, AAK97982, BAF40763, BAD06078, AAK97958, BAD11860, BAF40757, BAD06162, AAM89321, BAD11854, AAK97928, BAD11830, BAF40775, BAB11704, BAC81928, AAK97988, BAD11902, BAD11824, BAD06233, BAC81886, BAC81880, AAM89345, BAD06168, AAM89333, BAD06132, BAC82365, AAK97952, BAA01964, BAA01963, AAK97934, BAD06114, AAK97976, BAD21277, AAR13077, BAE02908, AAR12957, AAR02463, AAR02457, BAE03058, AAR89235, BAE02896, AAR89241, BAE02890, BAE03064, BAE03070, BAE03082, AAG34673, AAG34667, AAR89205, AAR89217, AAR13659, BAE03088, BAE03160, AAQ88264, AAR89187, AAR89283, AAK28472, AAR06661, AAR89253, AAR89247, AAR89199, AAR89193, AAR89229, AAR89223, AAR89265, AAR32743, AAR89277, BAE03166, BAE02920, BAE02914, BAE03112, BAE03106, BAE03100, BAE03094, BAE03076, BAE02944, BAE02998, BAE02992, BAE02986, BAE02980, BAE02974, BAE02968, BAE02962, BAE03016, BAE02902, BAE03040, AAR89211, AAR89271, BAE02956, BAE02938, BAE02932, BAE03148, BAE02950, BAE03004, BAE03154, BAE03142, BAE03136, BAE03130, BAE03124, BAE03118, BAE02926.

## Supporting Information

Figure S1 Electron micrographs of Virus Like Particles (VLP) used in hemagglutination assay. Purified VLP samples were placed on carbon grids, briefly washed in water and negatively stained with uranyl acetate and allowed to dry. The grids were viewed and imaged on a Technai G2 Spirit BioTWIN TEM.electron microscope. The magnification bar represents 100 nm.
(0.43 MB TIF)

Table S1 JCV VP1 sequences from PML patients.
(0.34 MB DOC)

Table S2 Amino acid variability of JCV VP1 sequences.
(0.11 MB DOC)

Table S3 Amino acid variability of JCV VP1 sequences between VLPs.
(0.03 MB DOC)
