# Supplementary figures and images for: Adaptive Mutations in the JC Virus Protein Capsid Are Associated with Progressive Multifocal Leukoencephalopathy (PML)
Source: PLoS Genet. 2009 Feb 6;5(2):e1000368. doi: 10.1371/journal.pgen.1000368 (PMC2629573; doi:10.1371/journal.pgen.1000368)

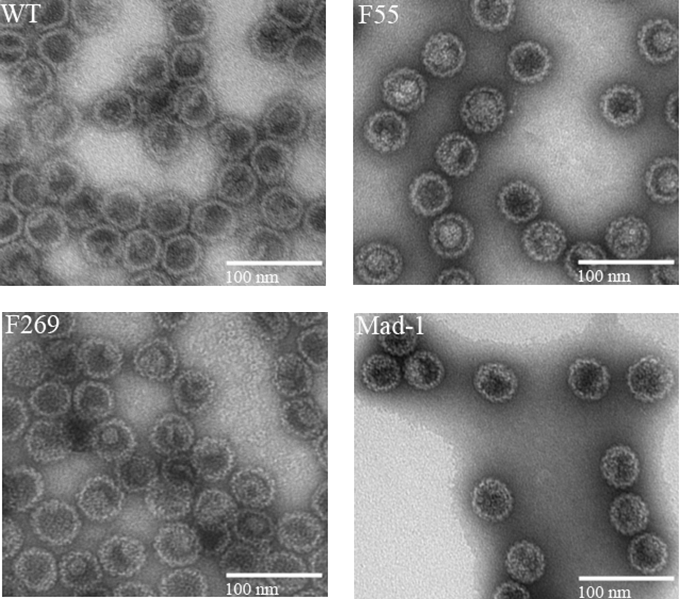

Supplement: Figure S1 — Electron micrographs of Virus Like Particles (VLP) used in hemagglutination assay. Purified VLP samples were placed on carbon grids, briefly washed in water and negatively stained with uranyl acetate and allowed to dry. The grids were viewed and imaged on a Technai G2 Spirit BioTWIN TEM.electron microscope. The magnification bar represents 100 nm. (0.43 MB TIF) [file pgen.1000368.s001.tif]
