# Supplementary material for: Adaptive Mutations in the JC Virus Protein Capsid Are Associated with Progressive Multifocal Leukoencephalopathy (PML)
Source: PLoS Genet. 2009 Feb 6;5(2):e1000368. doi: 10.1371/journal.pgen.1000368 (PMC2629573; doi:10.1371/journal.pgen.1000368)
Supplement: Table S1 — JCV VP1 sequences from PML patients. (0.34 MB DOC) [file pgen.1000368.s002.doc]

# Supplementary Table S1. JCV VP1 sequences from PML patients.

| N | DNA accession# | Protein accession# | DNA Source | Isolate name | AA length, | Start AA | Patient # | Reference |
| --- | --- | --- | --- | --- | --- | --- | --- | --- |
| 1 | AF015537 | AAB94036 | brain | 601 | 354 | 1 | 1 | [42] |
| 2 | AB183539 | BAE00111 | brain | 1-1 | 354 | 1 | 2 | [8] |
| 3 | AB183540 | BAE00117 | brain | 1-2 | 354 | 1 | 2 | [8] |
| 4 | AB183541 | BAE00123 | brain | 1-3 | 354 | 1 | 2 | [8] |
| 5 | AB183542 | BAE00129 | brain | 2-1 | 354 | 1 | 3 | [8] |
| 6 | AB183543 | BAE00135 | brain | 2-2 | 354 | 1 | 3 | [8] |
| 7 | AB183544 | BAE00141 | brain | 2-3 | 354 | 1 | 3 | [8] |
| 8 | AB190449 | BAE00147 | brain | 3-1 | 354 | 1 | 4 | [8] |
| 9 | AB190453 | BAE00171 | brain | 3-5 | 354 | 1 | 4 | [8] |
| 10 | AB190452 | BAE00165 | brain | 3-4 | 354 | 1 | 4 | [8] |
| 11 | AB190451 | BAE00159 | brain | 3-3 | 354 | 1 | 4 | [8] |
| 12 | AB190450 | BAE00153 | brain | 3-2 | 354 | 1 | 4 | [8] |
| 13 | AY536239 | AAT09819 | CSF | SA21_01 | 354 | 1 | 5 | [43] |
| 14 | AB212952 | BAE94726 | brain | ac-1 | 354 | 1 | 6 |  |
| 15 | AB212953 | BAE94732 | brain | ac-2 | 354 | 1 | 7 |  |
| 16 | D26589 | BAA05636 | brain | Aic-1a | 354 | 1 | 8 | [44] |
| 17 | AF004349 | AAB62680 | kidney | GS/K | 354 | 1 | 9 | [6] |
| 18 | AF004350 | AAB62687 | brain | GS/B | 354 | 1 | 9 | [6] |
| 19 | D11365 | BAA01967 | brain | Her1-Br | 354 | 1 | 10 | [45] |
| 20 | AB214923 | BAE02848 | CSF | JVL-10 | 245 | 39 | 11 | [9] |
| 21 | AB214924 | BAE02849 | CSF | JVL-11 | 245 | 39 | 12 | [9] |
| 22 | AB214925 | BAE02850 | CSF | JVL-12 | 245 | 39 | 13 | [9] |
| 23 | AB214926 | BAE02851 | CSF | JVL-13 | 245 | 39 | 14 | [9] |
| 24 | AB214927 | BAE02852 | CSF | JVL-16 | 245 | 39 | 15 | [9] |
| 25 | AB214928 | BAE02853 | CSF | JVL-17 | 245 | 39 | 16 | [9] |
| 26 | AB214929 | BAE02854 | CSF | JVL-18 | 245 | 39 | 17 | [9] |
| 27 | AB214930 | BAE02855 | CSF | JVL-19 | 245 | 39 | 18 | [9] |
| 28 | AB214912 | BAE02837 | brain | JVL-1a | 245 | 39 | 19 | [9] |
| 29 | AB214913 | BAE02838 | brain | JVL-1b | 245 | 39 | 19 | [9] |
| 30 | AB214914 | BAE02839 | brain | JVL-1c | 245 | 39 | 19 | [9] |
| 31 | AB214915 | BAE02840 | brain | JVL-1d | 245 | 39 | 19 | [9] |
| 32 | AB214916 | BAE02841 | brain | JVL-2 | 245 | 39 | 20 | [9] |
| 33 | AB214931 | BAE02856 | CSF | JVL-20 | 245 | 39 | 21 | [9] |
| 34 | AB214917 | BAE02842 | brain | JVL-3 | 245 | 39 | 22 | [9] |
| 35 | BAE02843 | BAE02843 | brain | JVL-4 | 245 | 39 | 23 | [9] |
| 36 | AB214919 | BAE02844 | brain | JVL-5 | 245 | 39 | 24 | [9] |
| 37 | AB214920 | BAE02845 | brain | JVL-7 | 245 | 39 | 25 | [9] |
| 38 | AB214921 | BAE02846 | brain | JVL-8 | 245 | 39 | 26 | [9] |
| 39 | AB214922 | BAE02847 | brain | JVL-9 | 245 | 39 | 27 | [9] |
| 40 | J02226 | AAA82101 | brain | Mad-1 | 354 | 1 | 28 | [46] |
| 41 | D11364 | BAA01966 | brain | Mad11-Br | 354 | 1 | 29 | [45] |
| 42 | D11363 | BAA01965 | brain | Mad8-Br | 354 | 1 | 30 | [45] |
| 43 | D11366 | BAA01968 | brain | NY-1B | 354 | 1 | 31 | [45] |
| 44 | AB212954 | BAE94738 | brain | oh-1 | 354 | 1 | 32 |  |
| 45 | AY536243 | AAT09843 | CSF | SA27_03 | 354 | 1 | 33 | [43] |
| 46 | AY536242 | AAT09837 | CSF | SA28_03 | 354 | 1 | 34 | [43] |
| 47 | AY536241 | AAT09831 | CSF | SA296_0 | 354 | 1 | 35 | [43] |
| 48 | AY536240 | AAT09825 | CSF | SA84_00 | 354 | 1 | 36 | [43] |
| 49 | D11367 | BAA01969 | brain | Sap-1 | 354 | 1 | 37 | [45] |
| 50 | D26590 | BAA05637 | brain | Tky-1 | 354 | 1 | 38 | [44] |
| 51 | AB038254 | BAB11728 | brain | Tky-1 | 354 | 1 | 39 | [10] |
| 52 | AB038255 | BAB11734 | brain | Tky-2a | 354 | 1 | 40 | [10] |
| 53 | D26591 | BAA05638 | brain | Tky-2a | 354 | 1 | 41 | [44] |
| 54 | D11368 | BAA01970 | brain | Tokyo-1 | 354 | 1 | 42 | [45] |
| 55 | AF030085 | AAC40846 | brain | Tokyo-1? | 354 | 1 | 43 | [47] |
| 56 | U21840 | AAB60586 | brain |  | 133 | 219 | 44 | [48] |
| 57 | U21839 | AAB60584 | brain |  | 133 | 219 | 45 | [48] |
| 58 | NA | NA | CSF | P9VP1 | 136 | 11 | 46 | [34] |
| 59 | NA | NA | CSF | P8VP1 | 136 | 11 | 47 | [34] |
| 60 | NA | NA | CSF | P7VP1 | 136 | 11 | 48 | [34] |
| 61 | NA | NA | CSF | P5VP1 | 136 | 11 | 49 | [34] |
| 62 | NA | NA | CSF | P4VP1 | 136 | 11 | 50 | [34] |
| 63 | NA | NA | CSF | P2VP1 | 136 | 11 | 51 | [34] |
| 64 | NA | NA | CSF | P1VP1 | 136 | 11 | 52 | [34] |
| 65 | NA | NA | CSF | P12VP1 | 136 | 11 | 53 | [34] |
| 66 | NA | NA | CSF | P11VP174 | 136 | 11 | 54 | [34] |
| 67 | NA | NA | CSF | P11VP173 | 136 | 11 | 54 | [34] |
| 68 | NA | NA | CSF | P11VP172 | 136 | 11 | 54 | [34] |
| 69 | NA | NA | CSF | P10VP1 | 136 | 11 | 55 | [34] |
