# Supplementary material for: Adaptive Mutations in the JC Virus Protein Capsid Are Associated with Progressive Multifocal Leukoencephalopathy (PML)
Source: PLoS Genet. 2009 Feb 6;5(2):e1000368. doi: 10.1371/journal.pgen.1000368 (PMC2629573; doi:10.1371/journal.pgen.1000368)
Supplement: Table S2 — Amino acid variability of JCV VP1 sequences. (0.11 MB DOC) [file pgen.1000368.s003.doc]

# Supplementary Table S2. Amino acid variability of JCV VP1 sequences

| **Position** | **Reference** | **Sequences with substitutions, (total)** | **% sequences with substitutions** | **Mutation (s)** | **Sequences with substitutions, (total)** | **% sequences with substitutions** | **Mutation (s)** |
| --- | --- | --- | --- | --- | --- | --- | --- |
| **8** | **G** | **4 (253)** | **2** | **4A** | **0 (32)** | **0** |  |
| **37** | **I** | **8 (253)** | **3** | **8V** | **0 (64)** | **0** |  |
| **52** | **D** | **1 (253)** | **0** | **1N** | **0 (64)** | **0** |  |
| **55** | **L** | **0 (253)** | **0** |  | **10 (64)** | **16** | **10F** |
| **60** | **K** | **1 (253)** | **0** | **1R** | **4 (64)** | **6** | **2M 1E 1N** |
| **61** | **S** | **0 (253)** | **0** |  | **5 (64)** | **8** | **1L 4P** |
| **64** | **D** | **0 (253)** | **0** |  | **6 (64)** | **9** | **5H 1N** |
| **69** | **E** | **5 (253)** | **2** | **5D** | **2 (64)** | **3** | **2D** |
| **70** | **S** | **2 (253)** | **1** | **2G** | **0 (64)** | **0** |  |
| **74** | **N** | **6 (253)** | **2** | **6S** | **12 (64)** | **19** | **12S** |
| **75** | **K** | **2 (253)** | **1** | **2R** | **2 (64)** | **3** | **2R** |
| **107** | **T** | **0 (253)** | **0** |  | **1 (64)** | **2** | **1N** |
| **113** | **I** | **59 (253)** | **23** | **59L** | **18 (64)** | **28** | **18L** |
| **115** | **V** | **1 (253)** | **0** | **1E** | **0 (64)** | **0** |  |
| **117** | **T** | **23 (253)** | **9** | **23S** | **15 (64)** | **23** | **15S** |
| **123** | **S** | **0 (253)** | **0** |  | **3 (64)** | **5** | **3C** |
| **128** | **T** | **25 (253)** | **10** | **25A** | **18 (64)** | **28** | **18A** |
| **129** | **H** | **0 (253)** | **0** |  | **1 (64)** | **2** | **1R** |
| **131** | **N** | **1 (253)** | **0** | **1K** | **0 (64)** | **0** |  |
| **134** | **A** | **186 (253)** | **74** | **186G** | **59 (64)** | **92** | **59G** |
| **153** | **E** | **1 (253)** | **0** | **1K** | **0 (52)** | **0** |  |
| **158** | **V** | **5 (253)** | **2** | **5L** | **3 (52)** | **6** | **3L** |
| **164** | **T** | **177 (253)** | **70** | **176K 1M** | **48 (52)** | **92** | **48K** |
| **167** | **D** | **1 (253)** | **0** | **1H** | **0 (52)** | **0** |  |
| **171** | **F** | **1 (253)** | **0** | **1Y** | **0 (52)** | **0** |  |
| **185** | **E** | **1 (253)** | **0** | **1D** | **0 (52)** | **0** |  |
| **192** | **K** | **2 (253)** | **1** | **2E** | **0 (52)** | **0** |  |
| **223** | **V** | **0 (253)** | **0** |  | **1 (56)** | **2** | **1A** |
| **258** | **D** | **1 (253)** | **0** | **1Y** | **0 (56)** | **0** |  |
| **265** | **N** | **0 (253)** | **0** |  | **4 (56)** | **7** | **3D 1T** |
| **267** | **S** | **0 (253)** | **0** |  | **4 (56)** | **7** | **3F 1L** |
| **269** | **S** | **2 (253)** | **1** | **1A 1T** | **13 (56)** | **24** | **9F 3Y 1C** |
| **271** | **Q** | **0 (253)** | **0** |  | **1 (56)** | **2** | **1H** |
| **277** | **R** | **1 (253)** | **0** | **1K** | **0 (56)** | **0** |  |
| **287** | **R** | **1 (253)** | **0** | **1K** | **0 (56)** | **0** |  |
| **310** | **D** | **1 (253)** | **0** | **1N** | **0 (56)** | **0** |  |
| **320** | **Q** | **1 (253)** | **0** | **1H** | **0 (56)** | **0** |  |
| **321** | **I** | **134 (253)** | **53** | **134V** | **30 (34)** | **88** | **30V** |
| **332** | **Q** | **184 (253)** | **73** | **184E** | **31 (34)** | **91** | **31E** |
| **345** | **R** | **20 (253)** | **8** | **20K** | **6 (34)** | **18** | **6K** |

Residues highlighted in red are distinct between PML and non-PML groups and have Bayes Empirical Bayes posterior probability for positive selection >0.5 (**Table I**). Residues highlighted in green are distinct between PML and non-PML groups.
