# Supplementary material for: Adaptive Mutations in the JC Virus Protein Capsid Are Associated with Progressive Multifocal Leukoencephalopathy (PML)
Source: PLoS Genet. 2009 Feb 6;5(2):e1000368. doi: 10.1371/journal.pgen.1000368 (PMC2629573; doi:10.1371/journal.pgen.1000368)
Supplement: Table S3 — Amino acid variability of JCV VP1 sequences between VLPs. (0.03 MB DOC) [file pgen.1000368.s004.doc]

# Supplementary Table S3. Amino acid variability of JCV VP1 sequences between VLPs.

|  | 55 | 74 | 75 | 117 | 128 | 134 | 158 | 164 | 269 | 321 | 332 | 345 |
| --- | --- | --- | --- | --- | --- | --- | --- | --- | --- | --- | --- | --- |
| WT1  AAQ88264 | L | N | K | T | T | A | V | T | S | I | Q | R |
| 55F  AAT09831 | F | N | K | T | T | A | V | T | S | I | Q | R |
| WT2(Mad-1)  P03089 | L | N | R | S | T | G | L | K | S | V | E | K |
| 269F  BAE00117 | L | S | K | S | A | G | V | K | F | V | E | K |

Protein sequences were aligned using ClustalW, amino acids different in at least one sequence from the rest of sequences are shown with their positions indicated.
